# Supplementary material for: New Synthesized Activating Transcription Factor 3 Inducer SW20.1 Suppresses Resistin-Induced Metabolic Syndrome
Source: Biomedicines. 2023 May 23;11(6):1509. doi: 10.3390/biomedicines11061509 (PMC10295781; doi:10.3390/biomedicines11061509)
Supplement: Supplementary file 1 [file biomedicines-11-01509-s001.zip › biomedicines-2348195-supplementary.pdf]

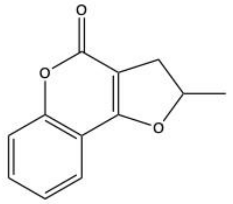

YHC06

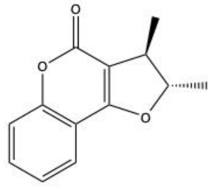

YHC08-1

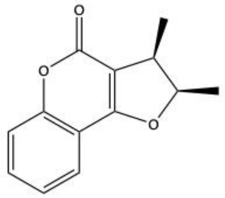

YHC08-2

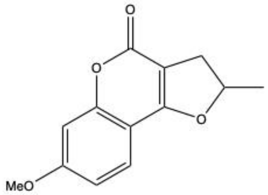

YHC15

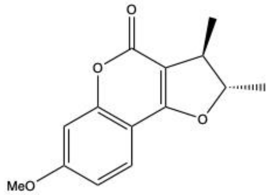

YHC16-1

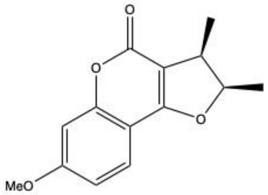

YHC16-2

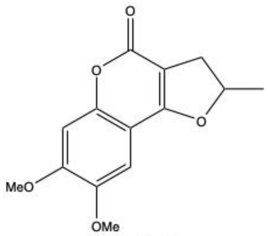

YHC25

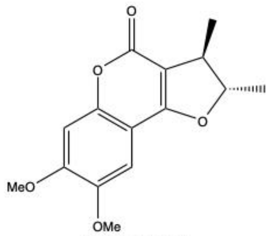

YHC29-1

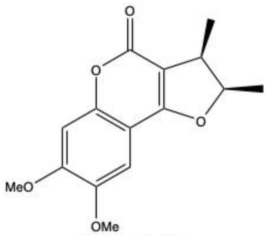

YHC29-2

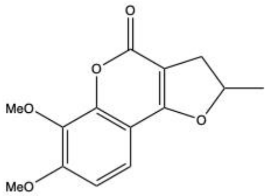

YHC27

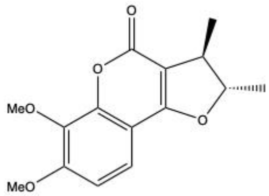

YHC28-1

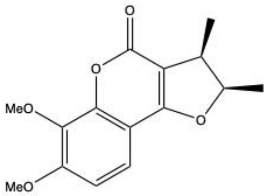

YHC28-2

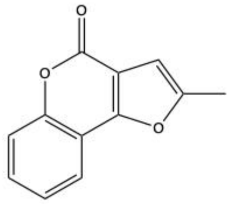

YHC18

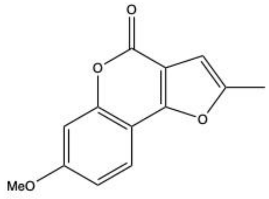

YHC19

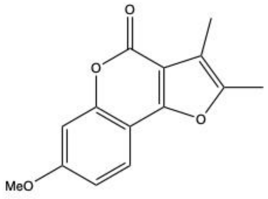

YHC21

Figure S1: The chemical structure of ATF3- inducer compounds

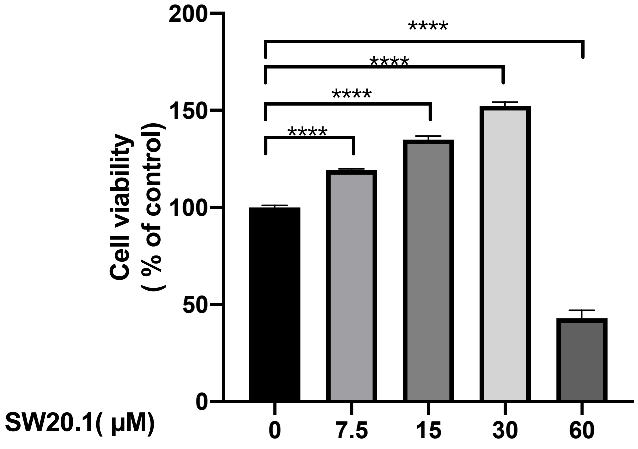

(a)

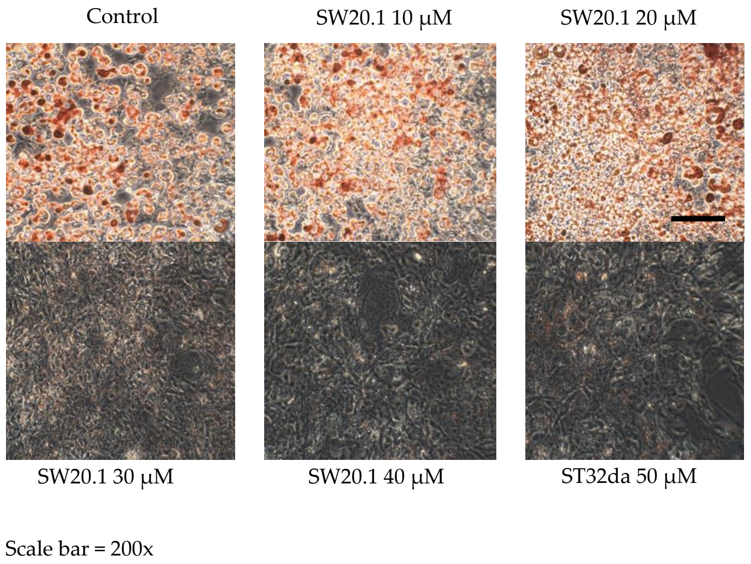

(b)

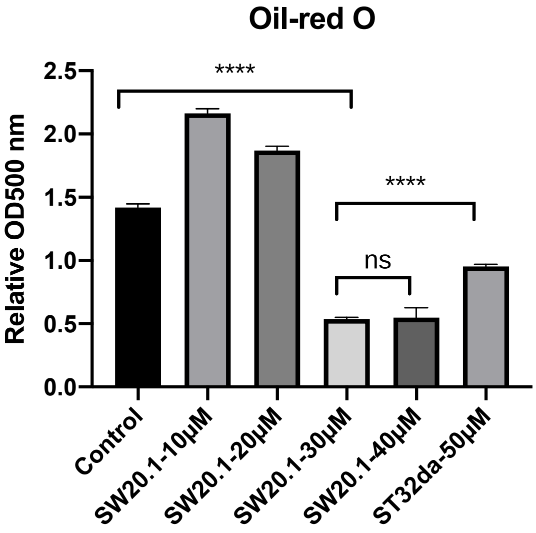

(c)

Figure S2. Effects of SW20.1 on cell viability in 3T3-L1 cell line and inhibited lipid accumulation of 3T3-L1 preadipocyte. (a) Cell viability was measured after treatment with various concentrations of SW20.1 (b) Oil red O staining of differentiated 3T3-L1 adipocytes treated with Vehicle, SW20.1, and ST32da for 8 days. (c) Relative qualification of the lipid content. Statistical comparisons were performed using unpaired t-test (a, c). Data are presented as mean ± standard error; **** $p < 0.0001$.

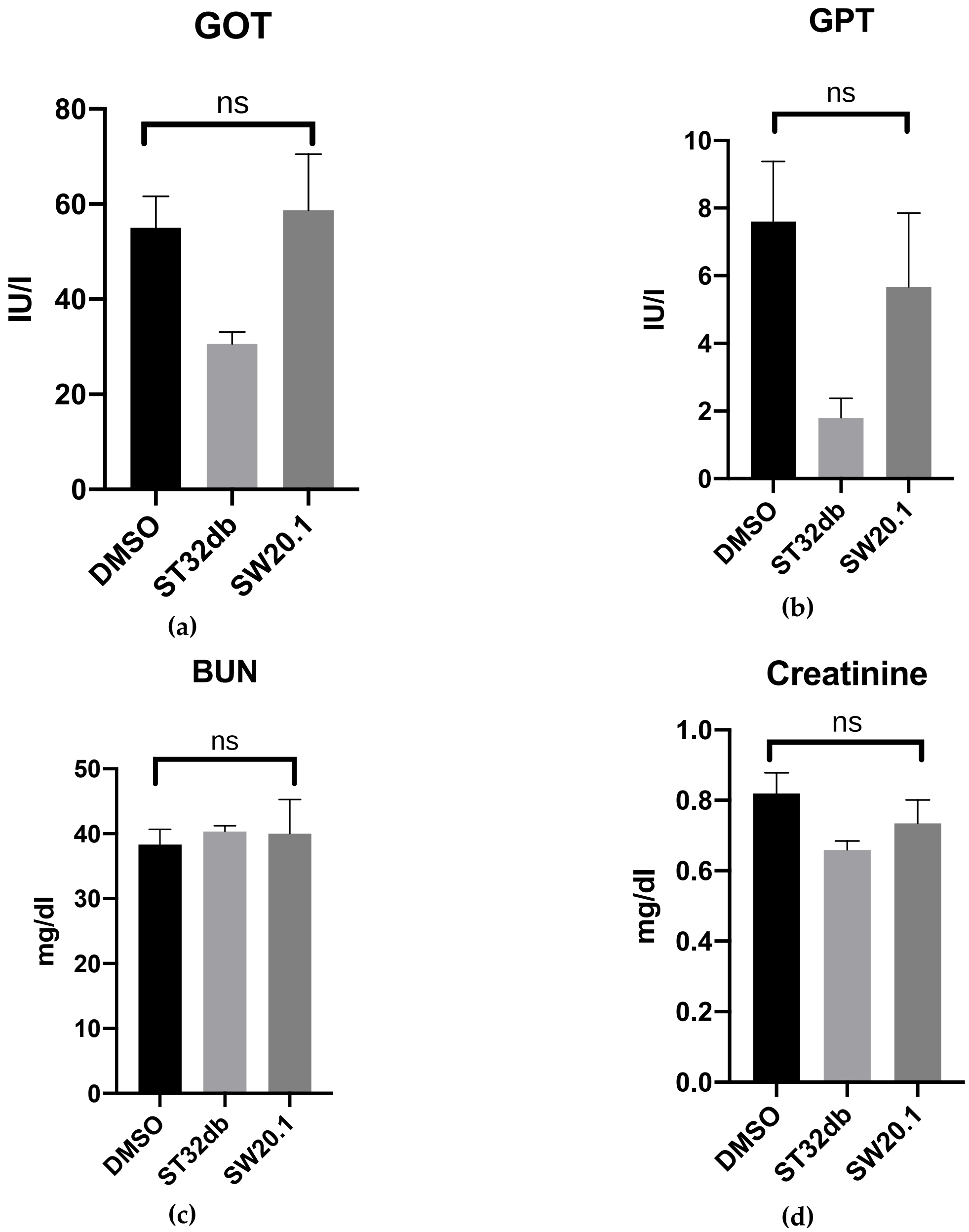

Figure S3: Biochemical parameters. (a) aspartate aminotransferase (GOT), (b) alanine aminotransferase (GPT),(c) Creatinin, (d) Blood Urea Nitrogen (BUN). Statistical comparisons were performed using unpaired t-test (a,b,c,d). Data are presented as mean ± standard error, ns non-significant.
